# Efficacy of Electroconvulsive Therapy for Comorbid Frontotemporal Dementia with Bipolar Disorder

**DOI:** 10.1155/2013/124719

**Published:** 2013-05-12

**Authors:** Sean Paul, Jennifer Goetz, Jeffrey Bennett, Tessy Korah

**Affiliations:** ^1^Department of Psychiatry, University of Florida, Gainesville, FL 32610, USA; ^2^Department of Radiology, University of Florida, Gainesville, FL 32610, USA

## Abstract

Challenges encountered in the diagnosis and treatment of frontotemporal dementia (FTD) are further confounded when presented with comorbid psychiatric disorder. Here we report a case of progressive FTD in a patient with a long history of bipolar affective disorder (BAD) 1, depressed type. We also report beneficial effects of electroconvulsive therapy and its potential application in similar comorbid disorders.

## 1. Introduction

Despite the emergence of frontotemporal dementia (FTD) as the second most common neurodegenerative cause of early onset dementia following Alzheimer's disease, its diagnosis and treatment are often problematic [1, 2]. The therapeutic and management options for FTD become even more challenging when presented with comorbid mood disorders such as bipolar affective disorder (BAD) 1, depressed type.

 In this report we describe a patient with a long history of BAD, further complicated by the diagnosis of FTD with emergence of novel behavioral and management issues. Presentation of catatonic-like symptoms further complicated the diagnosis and treatment options in this patient.

## 2. Case Report

The patient is a 51-year-old divorced college-educated male with a long history of bipolar affective disorder type 1 and subsequent treatment. He was working as a software engineer prior to the recent diagnosis of dementia NOS. At the time of the present hospitalization, he was transferred from a nursing home with severe dysphasia and recurrent choking episodes. The patient also was preoccupied with the delusion that he was the Archangel Michael.

At the age of 19, he was diagnosed with bipolar affective disorder type I, stabilized with *lithium,* and was able to lead a normal life until the age of 48 at which time his behavior started to show “bizarre” patterns. He began to display “childlike behaviors”—following his wife around the house and becoming increasingly argumentative. The patient was started on *methylphenidate* for unclear reasons and began experiencing difficulty completing complex computer codes; he also became increasingly argumentative and obstinate with his coworkers, which ultimately culminated in his dismissal. During this period of decline, the patient experienced manic episodes evidenced by racing thoughts, pressured speech, paranoid delusions, preoccupations with philosophy, religion, and some bizarre delusions. He began to purify his food and was convinced that someone was contaminating his contact lens solution. At some point during this period, *lithium*, which was a longstanding and very effective medication in this patient, was changed to *sodium valproate*. Despite the medication change, he continued to exhibit symptoms of psychosis coupled with persistent personality changes—manifested by verbal outbursts and lack of personal boundaries in social situations. 

Eighteen months prior to hospitalization, he was involuntarily committed for 6 weeks due to his inability to perform basic activities of daily living (ADL) and memory problems with ongoing psychosis. He came to our institution from an assisted living facility where he developed difficulty ambulating and dysphasia requiring medical hospitalization. Of note, at the nursing home he developed difficulty ambulating and dysphasia and was medically hospitalized only to be released without finding a medical cause. In between hospitalizations, it was noted that he was quite sensitive to low-dose risperidone. Although not physically aggressive, the patient continued to display repetitive and purposeless verbalizations, and paced vigorously. On arrival to our institution the patient was on *valproic acid, lithium, lorazepam*, *trazodone*, *risperidone, *and *haloperidol, *and continued to pace the halls and wander into patients' rooms. He came with reports of full neurological workup including MRI showing mild atrophy, CSF studies (AFB, gram stain, India ink, HIV and fungal studies) which were all negative, and EEG indicating diffuse background slowing with theta waves. The patient was admitted to our facility for treatment of a tentative diagnosis of bipolar affective disorder with psychotic and catatonic features. 

### 2.1. Mental Status Exam and Laboratory Tests

The patient was thin with disheveled hair and long facial hair and had overall poor grooming. The psychomotor slowing was prominent and he displayed purposeless repetitive mannerisms. He had significant speech delay and speech poverty often parroting back what was asked; he often responded inappropriately to questions. His affect was flat, and he laughed inappropriately throughout the interview. The overall thought process was disorganized and concrete with significant grandiose delusions and hyperreligiosity. He denied any homicidal or suicidal ideations and auditory or visual hallucinations and did not exhibit signs of internal preoccupation. His insight was superficial as he recognized he was sick and in a hospital but was unaware of the severity of his illness. His judgment, attention, and concentration were poor. MMSE was 6/28 (the patient was unable to draw; thus, copying objects and writing a sentence were omitted). Neuropsychological testing failed to yield relevant data as the patient could not sit still for a period of greater than several minutes. 

**Figure 1:**
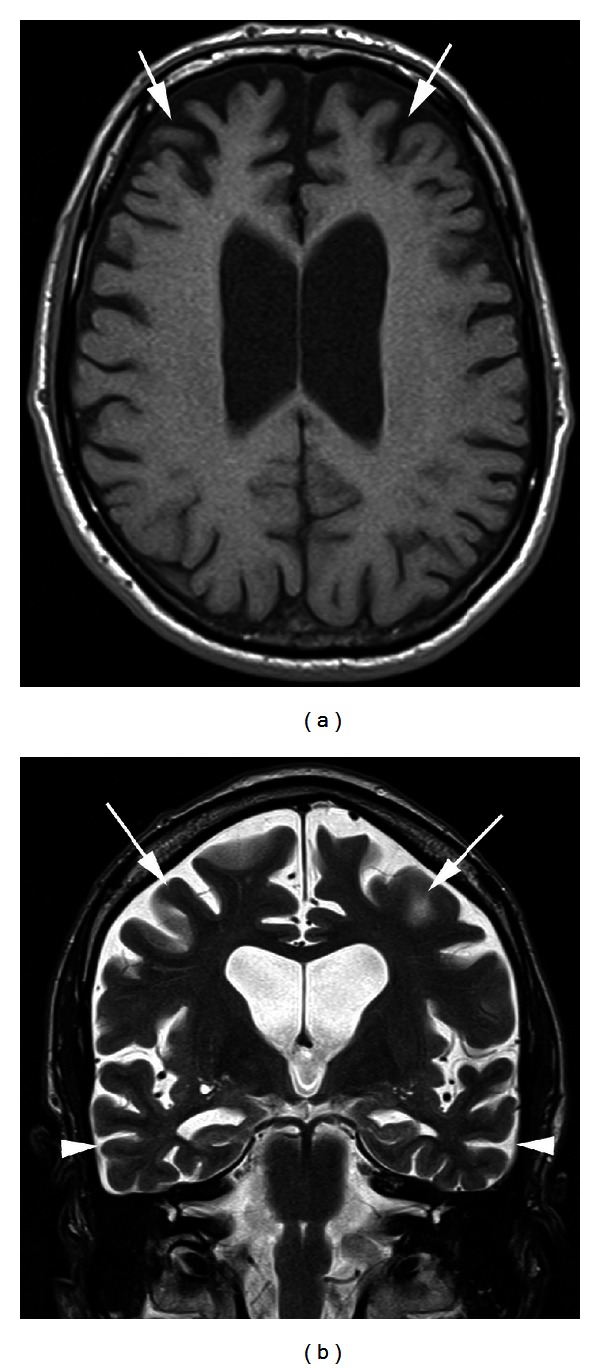
(a) An axial T1-weighted MR image of the brain demonstrating severe bilateral frontal lobe atrophy (arrows) compared to the parietal lobes. (b) A coronal T2-weighted MR image demonstrating severe bilateral frontal lobe atrophy (arrows) as well as bilateral temporal lobe atrophy (arrowheads).

The patient's complete blood count, comprehensive metabolic panel, B12, ammonia, and TSH levels were normal. CT of the head showed mild atrophy out of proportion to his age. MRI of the head showed age disproportionate volume loss with slightly more atrophy in the temporal and frontal regions. Prior imaging was reviewed and indicated no interval change over the 12 months separating the exams. The findings corresponded with a clinical diagnosis of frontotemporal dementia (see Figure 1). 

### 2.2. Hospital Course

After admission, the patient was continued on *valproic acid (500 mg twice a day) and lorazepam *(6 mg/day), which was tapered off gradually. Two weeks after admission, the patient began an ECT treatment regimen receiving nine treatments over the course of 5 weeks. The patient became verbally more responsive and interactive. Although he continued to have profound speech delay and word-finding difficulty, his MMSE significantly improved to 20/28 (patient could not complete the drawing and written portions of the exam). Despite improvements in his repetitive speech pattern, the wandering behavior and inappropriate laughter continued. His affect brightened and he reported a good mood. He was able to sit still, eat dinner, and watch a television program, things he was previously unable to do in the assisted living facility. The patient was discharged on *Depakote* 500 mg twice daily with a therapeutic blood level.

## 3. Discussion

Here we present the case of a challenging, rapid, and debilitating deterioration of a previously stabilized mood disorder. After multiple hospitalizations and complex medical regimens, a diagnosis of FTD with underlying BAD was made based on clinical, laboratory, and imaging data. Combined neurodegenerative and depressive disorder symptoms falsely presented catatonic features which complicated the diagnosis further [3, 4]. 

In the absence of definitive structural or functional brain imaging data, the diagnosis of FTD is generally difficult. Moreover, ambiguous clinical symptoms presented by this neurodegenerative syndrome often lead to misdiagnosis [5]. This patient also posed an additional challenge by not being able to complete neuropsychological testing due to profound distractibility and inattention. The symptoms of frontal behavioral disturbances and deterioration of language [6], combined with structural brain imaging data completed at an interval of one year, gave us a clear indication of comorbidity of FTD with BAD which was diagnosed almost 30 years earlier. However, our treatment options were limited due to the rapid deterioration of clinical features of BAD with a resultant poor MMSE score of 6/28. 

Faced with the difficulty of discerning the role of FTD and BAD in the clinical symptoms that appeared to be unresponsive to medications, an empirical regimen of ECT was used. Repeated studies have shown the efficacy of ECT on unipolar as well as bipolar depression [7, 8]. ECT has also been shown effective in a case of catatonia resembling FTD [9]. The confirmation of FTD based on post-ECT MRI further strongly discredits a potential role for catatonia in this patient. Despite the persistence of speech delay, word-finding difficulty, wandering behavior, and inappropriate laughter, the ECT regimen did resolve his affect and mood symptoms significantly. He was able to sit still to eat and watch television. Most significantly, his MMSE score went up to 20/28 from 6/28 before ECT. We were also able to taper off many of the medications without obvious impacts. Overall, the ECT appeared to have a beneficial effect on this patient with a very rare comorbid condition. 

Recent studies suggest that successful ECT can resolve MDD-associated impairments in the connectivity in frontal regions [10]. In comorbid conditions where both FTD and BAD have impacts on the frontal regions, the significant improvement in MMSE in this patient is due to a resolution of FTD or BAD effects, needs to be addressed by further brain imaging and molecular studies.
